# Blacklists and Whitelists To Tackle Predatory Publishing: a Cross-Sectional Comparison and Thematic Analysis

**DOI:** 10.1128/mBio.00411-19

**Published:** 2019-06-04

**Authors:** Michaela Strinzel, Anna Severin, Katrin Milzow, Matthias Egger

**Affiliations:** a Swiss National Science Foundation, Bern, Switzerland; b Institute of Social and Preventive Medicine (ISPM), University of Bern, Bern, Switzerland; American Society for Microbiology

**Keywords:** journal whitelists and blacklists, open access, peer review, predatory publishing, publishing ethics, scholarly communication, transparency

## Abstract

We aimed to develop an in-depth understanding of quality criteria for scholarly journals by analyzing journals and publishers indexed in blacklists of predatory journals and whitelists of legitimate journals and the lists’ inclusion criteria. To quantify content overlaps between blacklists and whitelists, we employed the Jaro-Winkler string metric. To identify topics addressed by the lists’ inclusion criteria and to derive their concepts, we conducted qualitative coding. We included two blacklists (Beall’s and Cabells Scholarly Analytics’) and two whitelists (the Directory of Open Access Journals’ and Cabells Scholarly Analytics’). The number of journals per list ranged from 1,404 to 12,357, and the number of publishers ranged from 473 to 5,638. Seventy-two journals and 42 publishers were included in both a blacklist and a whitelist. Seven themes were identified in the inclusion criteria: (i) peer review; (ii) editorial services; (iii) policy; (iv) business practices; (v) publishing, archiving, and access; (vi) website; and (vii) indexing and metrics. Business practices accounted for almost half of the blacklists’ criteria, whereas whitelists gave more emphasis to criteria related to policy. Criteria could be allocated to four concepts: (i) transparency, (ii) ethics, (iii) professional standards, and (iv) peer review and other services. Whitelists gave most weight to transparency. Blacklists focused on ethics and professional standards. Whitelist criteria were easier to verify than those used in blacklists. Both types gave little emphasis to quality of peer review. Overall, the results show that there is overlap of journals and publishers between blacklists and whitelists. Lists differ in their criteria for quality and the weight given to different dimensions of quality. Aspects that are central but difficult to verify receive little attention.

## Abstract (precis)

Predatory journals are spurious scientific outlets that charge fees for editorial and publishing services that they do not provide. Their lack of quality assurance of published articles increases the risk that unreliable research is published and thus jeopardizes the integrity and credibility of research as a whole. There is increasing awareness of the risks associated with predatory publishing, but efforts to address this situation are hampered by the lack of a clear definition of predatory outlets. Blacklists of predatory journals and whitelists of legitimate journals have been developed but not comprehensively examined. By systematically analyzing these lists, this study provides insights into their utility and delineates the different notions of quality and legitimacy in scholarly publishing used. This study contributes to a better understanding of the relevant concepts and provides a starting point for the development of a robust definition of predatory journals.

## INTRODUCTION

There is increasing concern in the scientific community and society about “predatory” journals, also called fake, pseudo-, or fraudulent journals. These allegedly scholarly open-access (OA) publishing outlets employ a range of unethical publishing practices; despite claiming otherwise and charging for it, they do not provide editorial services and scientific quality control. Predatory journals thus exploit the OA model of publishing; they generate revenue by requiring authors or their institutions to pay article-processing charges (APCs). There is widespread agreement that fraudulent journals pose a threat to the integrity of scholarly publishing and the credibility of academic research (1–7).

There have been various attempts to derive criteria to characterize and identify predatory journals, in order to help researchers to avoid these outlets (8). These attempts include the compilation of lists of fraudulent journals (blacklists) or trustworthy journals (whitelists). The best-known list is the blacklist of “potential, possible, or probable predatory scholarly open-access journals” by Jeffrey Beall, a librarian who coined the term “predatory” journal in 2015 (herein referred to as Beall’s list) (9). Beall took his list down in 2017, probably due to lawsuits instigated by publishers included in the list (7). At present, the list is maintained and updated by an anonymous scholar at a different site (10). While blacklists aim to alert authors to presumed fraudulent journals, whitelists take the inverse approach of providing an index of vetted, presumably legitimate outlets. Whitelists can differ substantially in their governance structures and funding models. While some whitelists require subscription or membership fees from journals or publishers, others are independent of publishers. The selection of journals for inclusion in blacklists and whitelists is based on a set of criteria which a journal has to meet in order to be included. Predominantly, whitelist criteria refer to proficiency and adherence to best practices to confirm the legitimacy of a journal. In the case of blacklists, these criteria describe undesirable, unethical, and deceptive practices that are believed to characterize fraudulent journals (11). As such, the two types of lists present different perspectives on the same challenge, ensuring the quality and legitimacy of academic publishing practices. Approaches other than blacklists and whitelists include decision trees or checklists to help authors distinguish between fraudulent and legitimate journals, for example, Think.Check.Submit. (1, 12, 13).

Despite the ongoing discussions on fraudulent publishing and the growing body of research on its market characteristics and prevalence, the defining attributes of fraudulent, illegitimate journals remain controversial (14, 15). Given that the prevalence of predatory journals can be assessed only if their assessment is based on a clear definition of fraudulent publishing, systematic studies on the understanding of quality and legitimacy in academic publishing are needed. This study aims to contribute to a better understanding of prevalent notions of good and poor quality in academic publishing by analyzing the inclusion criteria and journals and publishers included in blacklists of fraudulent journals and whitelists of legitimate journals.

(This article was submitted to an online preprint archive [16].)

## RESULTS

Two blacklists, the updated list of Beall (10) and Cabells Scholarly Analytics’ blacklist (17) (herein called Cabell’s blacklist), and two whitelists, the Directory of Open Access Journals (DOAJ; https://doaj.org/, accessed 2 December 2018) and Cabells Scholarly Analytics’ whitelist (17) (herein called Cabell’s whitelist), met our inclusion criteria. A subscription to the lists of Cabells Scholarly Analytics was purchased for this study, whereas access to the DOAJ and the updated Beall’s list was free of charge. While Beall’s list and the DOAJ are limited to OA journals and publishers, Cabell’s lists cover both OA and closed-access journals and publishers. Beall’s list included the fewest journals, but unlike the other three lists, Beall’s list contains two separate lists of journals and publishers, which are independent of one another. Journals included in Beall’s list of “standalone journals” are not linked to the publishers listed in Beall’s list of publishers. For this reason, we analyzed the lists’ contents separately for journals and publishers. Table 1 summarizes the features of the included lists.

**TABLE 1:** Characteristics of blacklists and whitelists included in the study

List	Maintenance	Access	Type(s) of journals and publishers	No. of journals	No. of publishers	Inclusion criteria used in analysis
Blacklists						
Beall’s list^*a*^	Formerly performed by an individual scholarly librarian; now performed by an academic wishing to remain anonymous	Free	Standalone OA journals and OA publishers	1,404	1,205	54 criteria developed by Jeffrey Beall, based on statements from the COPE and WAME (http://www.wame.org/)
Cabell’s blacklist	Employees of a for- profit company	Subscription	OA and subscription- based journals and publishers (ratio, 3:1)	10,671	473	63 criteria
Whitelists						
Cabell’s whitelist	Employees of a for- profit company	Subscription	OA and hybrid or subscription- based journals and publishers (ratio, 1:4)	11,057	2,446	38 criteria, not including criteria defining which disciplines are allowed in the list
DOAJ	Community of OA publishers with >100 volunteers and a core team of 15 people, employed by DOAJ's holding company, IS4OA	Free	OA journals and publishers	12,357	5,638	10 basic inclusion criteria, 14 principles of transparency, 15 additional recommendations, not including OA-specific criteria

a Unlike the other lists, journals and publishers included in the two Beall’s lists are independent of each other. All lists were accessed on 13 December 2018.

### Quantitative analysis of contents.

**FIG 1:**
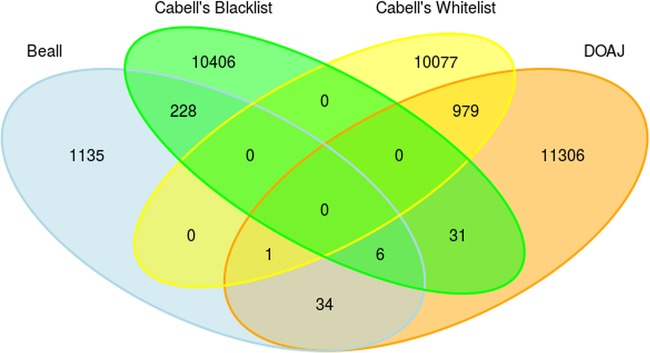
Venn diagrams of journal overlaps between Beall's list, Cabell's blacklist, the DOAJ, and Cabell’s whitelist (as of December 2018).

**FIG 2:**
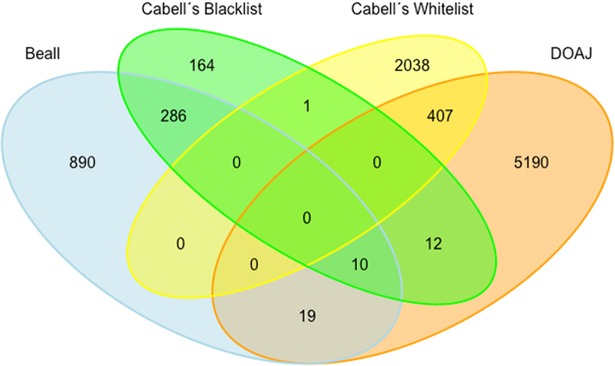
Venn diagram of publisher overlap between Beall's list, Cabell's blacklist, the DOAJ, and Cabell's whitelist (as of December 2018).

Table 2 shows the number of journals and publishers included in each list. For each pair of lists the number of matching journals and publishers and percent overlap are provided. As expected, there is considerable overlap between blacklists and blacklists and between whitelists and whitelists but also some overlap between whitelists and blacklists (Fig. 1 and 2).

**TABLE 2:** Cross-comparison of overlaps between blacklists and whitelists in this study

List	Category	No. (% overlap) of journals or publishers in^*a*^:			
		Beall’s list	Cabell’s blacklist	DOAJ	Cabell’s whitelist
Beall’s list	Journals	**1,404**	234 (16.7)	41 (2.9)	1 (0.07)
	Publisher	**1,205**	296 (24.6)	29 (2.4)	0 (0)
Cabell’s blacklist	Journals	234 (2.2)	**10,671**	37 (0.3)	0 (0)
	Publishers	296 (62.5)	**473**	22 (4.7)	1 (0.2)
DOAJ	Journals	41 (0.3)	37 (0.3)	**12,357**	980 (8)
	Publishers	29 (0.5)	22 (0.4)	**5,638**	407 (7.2)
Cabell’s whitelist	Journals	1 (0)	0 (0)	980 (8.9)	**11,057**
	Publishers	0 (0)	1 (0.04)	407 (16.6)	**2,446**

a Data are as of December 2018. Numbers in bold indicate the numbers of journals or publishers included in one list. Percentages indicate the proportions of journals or publishers in the supraindicated list also in the boldface number.

Overlap between blacklists was greater for publishers than for journals. Of all journals included in Beall's list and Cabell’s blacklist (*n* = 12,075), 234 journals were identical (1.9%), and of all publishers appearing in the lists (*n* =1,678), the overlap was 17.6% (*n* = 296). While the overlap of publishers accounted for only 16.6% of Beall’s list, it accounted for more than half of the publishers blacklisted by Cabells Scholarly Analytics (62.5%), Regarding the overlaps between the two whitelists, the percentages of journals and publishers that appeared on both the DOAJ and Cabell’s whitelist were 4.2% (*n* = 980) and 5.0% (*n* = 407), respectively.

Overlaps between Cabell’s whitelist and the two blacklists were small: only one journal that matched Beall’s list and one publisher that matched Cabell’s blacklist were found. In contrast, we identified more overlap between the DOAJ and the two blacklists. There were 41 journals (0.3% of 13,779 journals) and 29 publishers (0.4% of 6,843 publishers) that appeared in both the DOAJ and Beall’s list and 37 journals (0.2% of 23,046 journals) and 22 publishers (0.4% of 6,111 publishers) that were indexed in both the DOAJ and Cabell’s blacklist. Names of journals and publishers included in both types of lists are given in Table 3.

**TABLE 3:** List of names of journals and publishers included in a blacklist and a whitelist^*a*^

Journal (ISSN)
Journals included in Beall’s list, the DOAJ, and Cabell’s blacklist
Ecoforum (2344-2174)
European Chemical Bulletin (2063-5346)
Global Journal of Medicine and Public Health (2277-9604)
International Archives of Medicine (1755-7682)
International Journal of Mosquito Research (2348-7941)
Journal of New Sciences (2286-5314)


Journal included in Beall’s list, the DOAJ, and Cabell’s whitelist
International Journal of Nanomedicine (1178-2013)

Journals included in Beall’s list and the DOAJ
International Journal of Science, Culture and Sport (2148-1148)
International Review of Social Sciences and Humanities (2248–9010)
Journal of Advanced Veterinary and Animal Research (2311-7710)
Journal of Animal and Plant Sciences (2071-7024)
Journal of Arts and Humanities (2167-9045)
Journal of Clinical and Analytical Medicine (1309-0720)
Journal of Coastal Life Medicine (2309-5288)
Journal of Evidence Based Medicine and Healthcare (2349-2562)
Journal of HerbMed Pharmacology (2345-5004)
Journal of IMAB (1312-773X)
Journal of Intercultural Ethnopharmacology (2146-8397)
Journal of Media Critiques (2056-9793)
Jundishapur Journal of Health Sciences (2252-021X)
Junior Scientific Researcher (2458-0341)
Mediterranean Journal of Chemistry (2028-3997)
Mediterranean Journal of Modeling and Simulation (2335-1357)
OIDA International Journal of Sustainable Development (1923-6654)
Progress in Physics (1555-5534)
Tropical Plant Research (2349-1183)

Journals included in Cabell’s blacklist and the DOAJ
International Journal of Education and Literacy Studies (2202-9478)
International Journal of Pharmacological Research (2277-3312)
Journal of Education in New Century (2372-6539)
Journal of Men's Health (1875-6859)
Journal of Proteins and Proteomics (0975-8151)
Journal of Systemics, Cybernetics and Informatics (1690-4524)
Leonardo Electronic Journal of Practices and Technologies (1583-1078)
Leonardo Journal of Sciences (1583-0233)
Open Journal for Educational Research (2560-5313)
Open Journal for Sociological Studies (2560-5283)
Problems of Management in the 21st Century (2029-6932)
BJ Kines: National Journal of Basic & Applied Sciences (2231-6140)
Journal of Baltic Science Education (1648-3898)
Problems of Education in the 21st Century (1822-7864)
Problems of Psychology in the 21st Century (2029-8587)

Publishers included in Beall’s list, the DOAJ, and Cabell’s blacklist
Academia Publishing
AcademicDirect Publishing House
Atlas Publishing, LP
Australian International Academic Centre
ICTACT Journals
Insight Medical Publishing (OMICS International)
International Institute of Informatics and Systemics
Scholar Science Journals
Scientia Socialis
New Century Science Press

Publisher included in Cabell’s blacklist and Cabell’s whitelist
i-manager publications
Publishers included in Beall’s list and the DOAJ
AgiAl Publishing House
Eurasian Publications
Herald Scholarly Open Access
Hilaris
Ivy Union Publishing
Longdom Publishing
PiscoMed Publishing
Scholarly Research Publisher
Science and Education Centre of North America
Scientia Ricerca
Elewa BioSciences
International Foundation for Research and Development
International Academy of Ecology and Environmental Sciences
New Century Science Press LLC
EconJournals
Science Park Research Organization and Counselling LTD
Applied Science Innovations Private Limited
Frontiers Media S.A.
NobleResearch Publishers

Publishers included in Cabell’s blacklist and the DOAJ
B J Medical College
Innovative Journal Solutions
International Medical Society
Scientia Socialis
New Century Science Press
Atlas Publishing, LP
The Dougmar Publishing Group, Inc.
Australian International Academic Centre
International Institute of Informatics and Systemics
Academy of Business and Retail Management
Academia Publishing
Center for Open Access in Science
AcademicDirect Publishing House
Association of Educational and Cultural Cooperation Suceava from Stefan cel Mare University
Regional Institute of Health and Family Welfare
Deuton-X Ltd.
ASTES Publishers
Sunblo Learning Center
ICTACT Journals
Scholar Science Journals
Serials Publications/International Science Press
Insight Medical Publishing (OMICS International)

a Data are as of December 2018.

### Qualitative analysis of inclusion criteria: thematic analysis.

**FIG 3:**
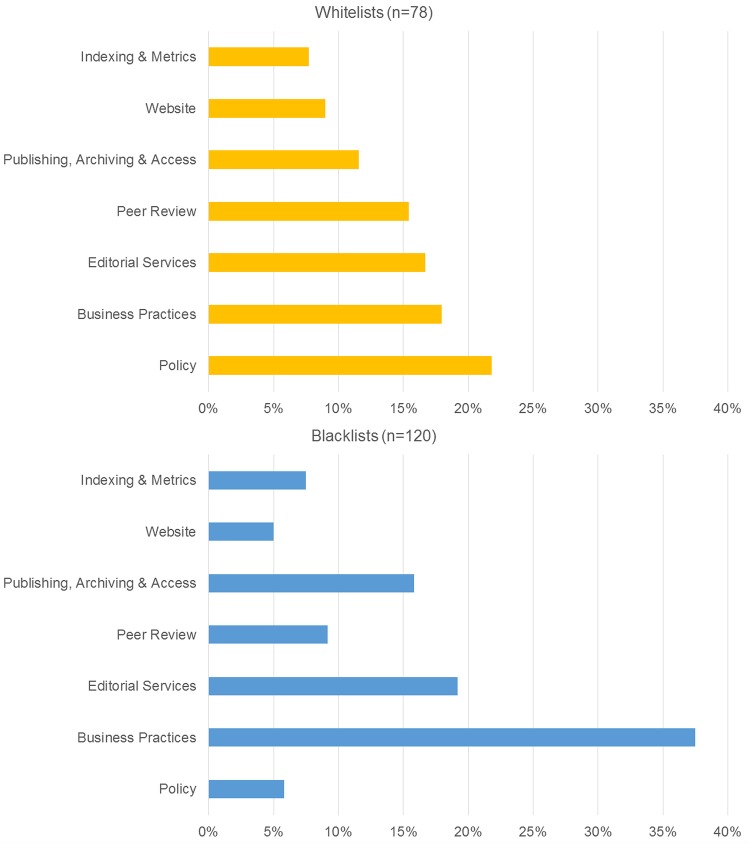
Distribution of inclusion criteria across seven thematic topics for whitelists and blacklists.

The analysis of inclusion criteria showed that some covered more than one criterion, and we therefore deconstructed these into separate criteria. A total of 198 criteria were finally included in the qualitative analysis, 120 from blacklists and 78 from whitelists (see Table S1 in the supplemental material). The iterative thematic analysis of the 198 criteria identified seven topics: (i) peer review; (ii) editorial services; (iii) policy; (iv) business practices; (v) publishing, archiving, and access; (vi) website; and (vii) indexing and metrics. The distribution of criteria across topics is summarized in Fig. 3 and Table 4 and discussed in detail below. Blacklists gave most emphasis to business practices, followed by editorial services and publishing practices, archiving, and access. For whitelists, policy was most extensively covered, followed by business practices and editorial services.

**TABLE 4:** Consolidated list of topics addressed by inclusion criteria for blacklists and whitelists

Topic (total no.)	Criteria included	No. of criteria (% within column) on:			
		Blacklists		Whitelists	
		Beall (*n* = 57)	Cabell (*n* = 63)	DOAJ (*n* = 40)	Cabell (*n* = 38)
Peer review (*n* = 23)	Presence/absence of peer review	6 (10.5)	5 (7.9)	4 (10.0)	8 (21.1)
	Type and quality of peer review				
	Qualifications of peer reviewers				

Policy (*n* = 24)	Presence/absence of author guidelines	4 (7.0)	3 (4.8)	9 (22.5)	8 (21.1)
	Presence/absence of policies regarding retraction, copyright/licensing, editorial services, peer review, etc.				

Business practices (*n* = 59)	Type of marketing activities	19 (33.3)	26 (41.3)	5 (12.5)	9 (23.7)
	Presence/absence of contact information				
	Type of or the presence/absence of information on the business model and legal status				
	Aspects of a journal’s self-representation, such as its name, mission, etc.				

Publishing, archiving, and access (*n* = 28)	Publishing practices, such as the main author and target group, the type of publication model, the type of literature published	7 (12.3)	12 (19.0)	4 (10.0)	5 (13.2)
	Access to the articles and information on access				
	Presence/absence of digital archives				

Website (*n* = 13)	Structure, functionality, grammar/spelling, advertisement, etc., of the website	3 (5.3)	3 (4.8)	6 (15.0)	1 (2.6)

Indexing and metrics (*n* = 15)	Presence/absence and respective authenticity of permanent journal identifiers (such as an ISSN or digital object identifier [DOI])	5 (8.8)	4 (6.3)	4 (10.0)	2 (5.3)
	Presence/absence of or type of journal metrics				

Editorial services (*n* = 36)	Presence/absence of, composition of, or information on the editorial board and editorial practices	13 (22.8)	10 (15.9)	8 (20.0)	5 (13.2)

10.1128/mBio.00411-19.1 TABLE S1 List of criteria by whitelist or blacklist, topic, concept, and verifiability. A list of the 198 criteria analyzed in this study, stratified by type of list, topic, concept, and verifiability is presented. Download Table S1, XLSX file, 0.04 MB. Copyright © 2019 Strinzel et al. 2019 Strinzel et al. This content is distributed under the terms of the Creative Commons Attribution 4.0 International license.

### (i) Peer review.

**FIG 4:**
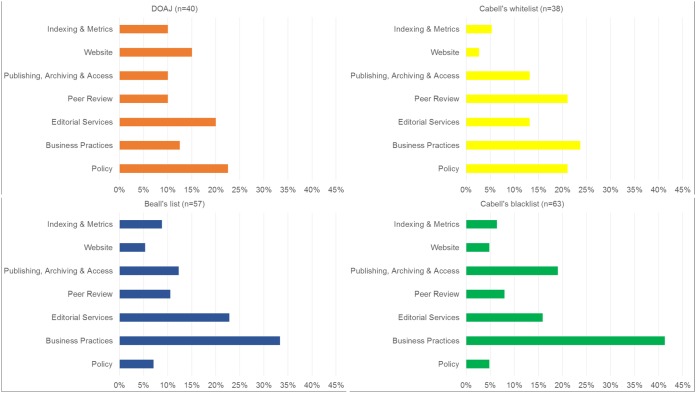
Distribution of inclusion criteria across seven thematic topics for the four lists.

Both blacklists and whitelists include criteria stating that a journal needs to have a “rigorous” peer review system in place (Table S1). Neither whitelist defines “rigorous”; however, Cabell Scholarly Analytics’ website states that peer review should be anonymous and conducted by at least two reviewers. The whitelists appear to rely on the information provided by the journal. Cabell’s whitelist also takes acceptance rates of a journal into account as a measure of selectivity. The criteria included in blacklists describe the peer review process as “insufficient,” “inadequate,” or “not bona fide” (Table S1). To judge the adequacy of peer review, blacklists make use of several indicators: the promise of fast publication, the acceptance of fake papers and obvious pseudoscience, publication of conference contributions without review, and the poor qualifications of reviewers. Beall considers reviewers unqualified if they lack expertise in the field that the journal covers or if the journal does not vet reviewers suggested by the author. With the exception of Cabell’s whitelist, the lists do not include many criteria referring to peer review. Figure 4 shows the distributions of topics for the four lists.

### (ii) Editorial services.

Regarding editorial services, both types of lists require an editorial board with qualified members, where “qualified” is defined as academic expertise in the journal’s field (Table S1). The lists require information on the board members’ names, their academic affiliations, and their contact details. The DOAJ particularly stresses this aspect (Fig. 4). In addition, blacklists consider the truthfulness of details about board members. Beall takes into account the number of board members (at least four [Table S1]). Other criteria of both Beall’s list and Cabell’s blacklist refer to the diversity of the editorial board in terms of geographical origin, gender, or ethnicity. Both blacklists address the lack of editorial services, such as copyediting and proofreading. They also consider whether the resources that a journal spends on preventing author misconduct are “sufficient,” with a focus on plagiarism. The whitelists value the use of plagiarism-screening tools. Criteria referring to the editorial services of a journal account for a relatively large proportion of criteria of the DOAJ and Beall’s list (Fig. 4).

### (iii) Policy.

Both blacklists and whitelists state that comprehensive policies should be in place, but they focus on different policies. Whitelists address aspects such as the presence of detailed author guidelines, with information on types of licensing, peer review and editorial services, handling of retractions, etc. In contrast, blacklists address the lack of policies on archiving, licensing, peer review, and author guidelines. Blacklists, moreover, focus on author guidelines, i.e., whether they are original or copied from another journal or of poor orthography. As shown in Fig. 4, the topic “policy” makes up the largest proportion of criteria of the DOAJ and many criteria in Cabell’s whitelist. The two blacklists, by contrast, contain only a few criteria on policy and guidelines.

### (iv) Business practices.

All lists address similar categories but do so to different degrees of detail. Blacklist criteria refer to the business model of a journal, its marketing activities (e.g., spam emails), and the way a journal promotes itself (e.g., boastful language). They also address the correctness of information about the location of the editorial office, legal status, management, and mission. The lack of membership in learned societies, the focus on profit (e.g., by offering prepay options) or the nondisclosure of the APC charged are all considered. Whitelists require unobtrusive marketing practices, contact details, and pricing transparency. Cabell’s whitelist, like the blacklists, considers membership in organizations like the Committee on Publication Ethics (COPE), the World Association of Medical Editors (WAME), and others. Both blacklists and Cabell’s whitelist put most weight on the business practices of a journal. For the DOAJ, this topic plays a less important role (Fig. 4).

### (v) Publishing practices, archiving, and access.

Blacklists assess the range of topics that a journal covers, whether its articles appear in more than one journal, and how easily articles can be accessed. In addition, multiple papers by the same authors in a journal are considered. Beall’s criteria refer to publications by the editor or lack of publications by members of the editorial board, both of which indicate bad publishing practices. Whitelist criteria are less specific and do not address authorship explicitly. Both types of lists state that articles should be permanently archived and easily accessible, irrespective of the type of access.

Whereas access to articles and publishing and archiving practices appear subordinate in Beall’s list, the DOAJ, Cabell’s whitelist, and Cabell’s blacklist include many criteria addressing these topics (Fig. 4).

### (vi) Website.

Both blacklists and whitelists are concerned with appearance and functionality of a journal’s website. Blacklists are more detailed and mention dead links, poor grammar and spelling, illegal use of copyrighted material, and cluttered and obtrusive advertising. Generally, aspects regarding the website of a journal are addressed by only a few criteria in both blacklists and whitelists. In relative terms, the DOAJ includes the highest number of criteria on this topic (Fig. 4).

### (vii) Indexing and metrics.

There is general agreement that a journal should have a permanent, verifiable identifier, such as an international standard serial number (ISSN). Moreover, being indexed in bibliographic databases is perceived as an indicator of a journal’s trustworthiness by both blacklists and Cabell’s whitelist. Whitelists, in particular, the DOAJ, stress that identifiers should be transparently displayed on a journal’s website. Regarding metrics, the DOAJ states that the prominent display of journal impact factors (JIFs) is bad practice. Blacklists, in contrast, check whether the information on metrics is correct and mention the use of fake metrics. If a JIF is mentioned, it should be the JIF of Thompson Reuters (now Clarivate Analytics). Indexing and metrics contribute a small proportion of the inclusion criteria for both blacklists and whitelists (Fig. 4).

### Qualitative analysis of inclusion criteria: conceptual analysis.

**FIG 5:**
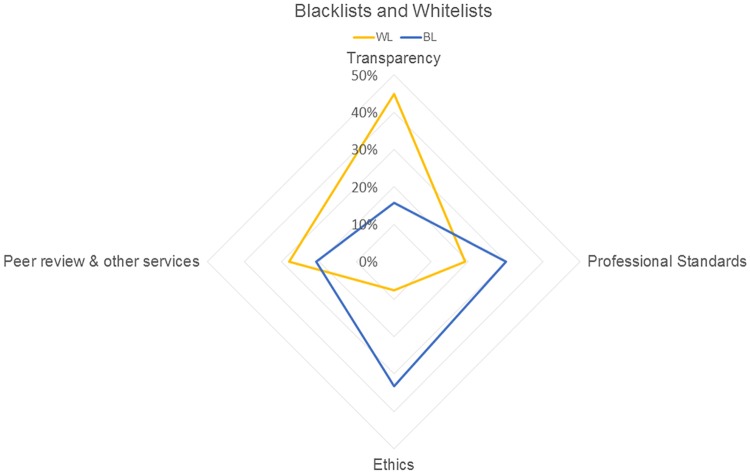
Distribution of inclusion criteria across four concepts for blacklists (BL) and whitelists (WL).

The analysis of criteria produced four concepts: (i) transparency, (ii) ethics, (iii) professional standards, and (iv) peer review and other services. Figure 5 shows the percentages of criteria of blacklists and whitelists that informed the different concepts. Compared to blacklists, whitelists gave more emphasis to transparency and less emphasis to professional standards and ethics. There were similar emphases on peer review and other services.

### (i) Transparency.

**FIG 6:**
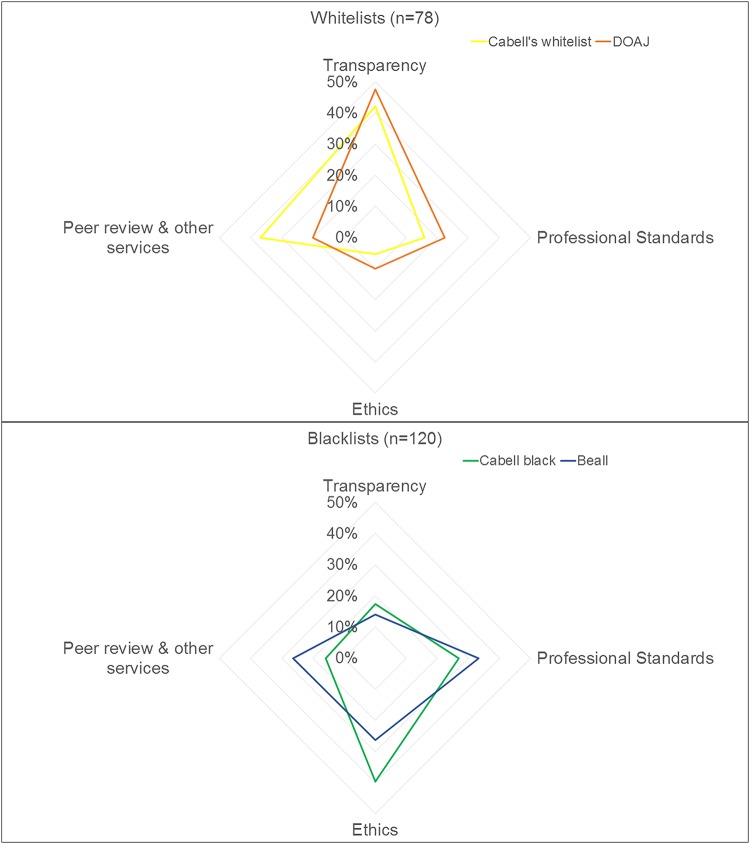
Distribution of inclusion criteria across four concepts for all four lists individually.

Criteria relating to transparency include the presence of guidelines and policies and transparent business and publishing practices. Whitelists address a broader range of topics than blacklists. For instance, both whitelists include a high number of criteria referring to the transparency of editorial practices, including, for example, the provision of names, affiliations, and contact details of the editorial board members (Table S1). The DOAJ includes the highest proportion of criteria related to transparency, whereas both blacklists have only a few criteria on this concept (Fig. 6).

### (ii) Ethics.

Criteria informing about business and publication ethics occupy much space in both blacklists. These criteria describe a range of unethical practices, from the provision of false or misleading information (regarding name, legal status, location, editorial board) and the use of fake metrics to unethical publishing practices (such as plagiarism). Cabell’s blacklist includes more criteria relating to ethics than Beall’s list (Fig. 6). Whitelists include only a few criteria on business ethics, most of which are general in nature. For example, the journal should not provide information that might mislead readers or authors (Table S1). Cabell’s whitelist considers whether a journal is a member of COPE or not. As mentioned above, the DOAJ includes the criterion that the prominent display of the impact factor is inappropriate.

### (iii) Professional standards.

This concept refers to a journal’s professional appearance and demeanor, as reflected by external features of a journal, such as its website and business practices (marketing activities and pricing). Professional standards are of central importance for blacklists, and in particular Beall’s list, but are less important for whitelists (see Fig. 6). Criteria related to the journal’s standing, such as whether it is indexed in a database or published by an association, are covered by both blacklists and Cabell’s whitelist.

### (iv) Peer review and other services.

This concept comprises criteria related to the provision of specific services, including peer review and editorial services and their quality. A small number of criteria address services such as the indexing of a journal in bibliographic databases, the long-term archiving of articles, and the protection against misconduct. Beall’s list and Cabell’s whitelist include more criteria related to the concept than the DOAJ and Cabell’s blacklist (Fig. 6). Criteria addressing editorial services focus mostly on the existence of an editorial board with qualified editors. Both blacklists and whitelists address peer review and its quality. The quality of a journal’s articles and the type of peer review (in terms of the duration of the review process or the number and qualification of reviewers) are used as proxies for quality in peer review (Cabell’s whitelist, Beall’s list, and Cabell’s blacklist). Guaranteeing acceptance or rapid publication is considered inappropriate by the DOAJ and regarded as a sign of poor peer review by both blacklists.

### Verifiability.

The verifiability of blacklist and whitelist criteria differed. The verifiability of inclusion criteria was easiest for the DOAJ and equally difficult for the three other lists (Table 5). In particular, the proportion of criteria categorized as easily verifiable through a single source was considerably greater for the DOAJ (77%) than for Beall’s list (31%) and both of Cabell’s lists (whitelist, 47%, and blacklist, 35%). The DOAJ includes a large number of criteria related to transparency, which are easier to assess than the other three concepts (Table 5). Cabell’s whitelist contains, besides a high number of easily verifiable criteria, many criteria that require individual judgment. These criteria often address peer review and editorial services. Items that require several sources for verification or prior contact with the journal are more common in blacklists and address predominantly professional standards as well as business and publishing ethics.

**TABLE 5:** Distribution of inclusion criteria across three levels of verifiability

List, topic, or concept (no.)	No. (%) verified when verifiability was:		
	Easy (one source required)	Intermediate (several sources required)	Difficult (subjective judgment required)
Lists (*n* = 198)			
DOAJ (*n* = 40)	31 (77)	4 (10)	5 (13)
Cabell’s whitelist (*n* = 38)	18 (47)	8 (21)	12 (31)
Beall’s list (*n* = 57)	18 (31)	25 (43)	14 (24)
Cabell’s blacklist (*n* = 63)	22 (35)	30 (48)	11 (17)
Total	89 (45)	67 (34)	42 (21)

Topics			
Peer review (*n* = 23)	7 (30)	3 (13)	13 (57)
Editorial services (*n* = 36)	14 (39)	10 (28)	12 (33)
Business practices (*n* = 59)	23 (39)	27 (46)	9 (15)
Policy (*n* = 24)	21 (88)	3 (14)	
Publishing, archiving, and access (*n* = 28)	9 (32)	12 (43)	7 (14)
Indexing and metrics (*n* = 15)	4 (15)	11 (73)	
Website (*n* = 13)	11 (84)	1 (8)	1 (8)

Concepts			
Transparency (*n* = 54)	48 (88)	4 (8)	2 (4)
Professional standards (*n* = 51)	24 (47)	23 (43)	5 (10)
Ethics (*n* = 46)	7 (15)	31 (67)	8 (18)
Peer review and other services (*n* = 47)	10 (21)	10 (21)	27 (48)

## DISCUSSION

This comprehensive study of blacklists of predatory journals and whitelists of legitimate journals triangulated quantitative and qualitative approaches. The qualitative analysis elucidated the multidimensional understanding of quality in academic publishing that underpins blacklists and whitelists. This multidimensionality is reflected at both the level of the specific topics addressed by criteria and the more abstract level of concepts. The thematic analysis of topics and concepts covered by the 198 inclusion criteria for the different lists resulted in seven topics and four broader concepts. It showed important differences between lists in the emphasis given to these topics: blacklists gave much emphasis to business practices, editorial services, and publishing practices. In contrast, whitelists covered policy most extensively, followed by business practices, editorial services, and peer review. Regarding the broader concepts, whitelists gave more emphasis to transparency and less emphasis to professional standards and ethics than blacklists. The two types of lists thus complement each other and contribute to a broader understanding of quality. Of note, the whitelist criteria were easier to verify than the criteria used by blacklists. In general, all lists appear to prioritize easily verifiable dimensions of a journal’s quality over the quality of scientific evaluation.

In the DOAJ, more criteria relate to transparency of business and publishing practices than to the quality of peer review. Studies have used transparency as a proxy of the quality of a journal (18). However, other studies have shown that statements made by a journal can be false and its transparency spurious (19). This indicates a risk of endorsing the legitimacy of a journal based on its transparent nature, while at the same time ignoring journals’ lack of best practices in peer review. Indeed, when John Bohannon, a science journalist at Harvard University, submitted a bogus scientific paper with major flaws to DOAJ-listed publishers, the weaknesses in their peer review were clearly exposed (20). Similarly, blacklist criteria predominantly relate to ethical issues and professional standards and not to the quality of the scientific evaluation of article submissions. Cabell’s whitelist appears more balanced in valuing different dimensions of journal quality, including peer review. The quality of peer review is difficult to evaluate. Standardized instruments have been used previously, for example, in the context of assessing the impact of open peer review (21, 22). Interestingly, publishers who were criticized for poor peer review and included in Beall’s list, such as MDPI or Frontiers, plan to make peer review reports openly accessible along with the article, so that readers can judge the thoroughness of its scientific evaluation. Evaluating the quality and thoroughness of scientific evaluation will require a sound definition of what constitutes “good” or “rigorous” peer review, but most standardized instruments appear to lack any theoretical foundation (23).

The quantitative analysis investigated overlaps between the contents of blacklists and whitelists. The considerable overlap between the two blacklists indicates that Cabell’s list may use Beall’s list as a source of predatory publishers. The relatively small overlap between the whitelists might be explained by the fact that the lists pursue different objectives regarding coverage. The overlaps that we found between blacklists and whitelists may be interpreted in several ways. First, these journals may be “false positives” on the blacklists, i.e., wrongly classified as fraudulent. Indeed, Beall’s list has been criticized for not distinguishing fraudulent from low-quality journals or from emerging journals, for example, journals from the global south. The latter may not be able to afford membership in associations or may not yet have been accepted as members and thus be misclassified by blacklists (24–26). Others have argued that even if describing undesirable practices, some of the criteria Beall used to characterize fraudulent journals are also applicable to established, presumably legitimate journals (27, 28). Second, these journals might be “false negatives” on the whitelists, i.e., wrongly classified as being legitimate, based on criteria that are easily verified and easily met but that miss other, fraudulent practices, for example, the lack of adequate peer review. Furthermore, the status of a journal may change over time, as publishers and editors abandon questionable practices or good practices. Lists therefore need to be kept up to date, and journals should be periodically reassessed. Third, some journals may operate in a gray zone for extended periods, meeting some blacklist and some whitelist criteria. Fourth, beside their common goal of identifying legitimate or illegitimate journals and publishers, the lists may follow other agendas, which might require a different weighing of inclusion criteria or might affect the inclusion or exclusion of certain journals and publishers. Although the overlap was small, the criteria in use for the different lists are unlikely to capture fully the quality and legitimacy in academic publishing. In other words, these lists can be useful, but they do not provide a completely accurate delimitation between legitimate and illegitimate journals. To gain a comprehensive understanding of the accuracy of lists, future studies could include additional lists in the analysis, such as the expanded version of Beall’s list (29, 30). We will examine the characteristics of journals that ended up on both blacklists and whitelists in detail in a follow-up study.

To our knowledge, this is the first systematic, comparative analysis of blacklists of predatory journals and whitelists of legitimate journals. A recent scoping review by Cobey and colleagues identified 109 characteristics of predatory journals, which were extracted from 38 empirical studies including a definition of predatory journals (31). In line with what we found for blacklist criteria, Cobey et al. report that most characteristics used to define predatory journals do not relate to the quality of the scientific evaluation of article submissions but rather to the journal’s business operations and revolve around the lack of transparency, integrity, and quality.

Our study has several limitations. First, we used Google to identify lists but may have missed some blacklists or whitelists. The results of Google searches differ across users in unpredictable ways and are not fully reproducible. Second, as fuzzy matching allows comparisons of strings on the basis of similarity rather than on a precise match, it is possible that we missed journals and publishers contained in both a blacklist and a whitelist. We downloaded the lists in December 2018. They therefore show a snapshot in time and might have changed since then. For instance, the overlap between the two Cabell lists reportedly was due to an internal system error by Cabells Scholarly Analytics and was rectified after we published the preprint (16). Third, qualitative analysis always entails a certain degree of subjectivity, as the assessor’s knowledge, background, and judgement influence data interpretation. To mitigate the subjective nature of data interpretation, two assessors analyzed the inclusion criteria. Fourth, in interpreting the criteria, we did not take into account potential list-specific weighting of criteria (the DOAJ has a hierarchy of criteria) but weighted all criteria equally for the sake of cross-list comparability. Finally, we restricted eligible blacklists and whitelists to interdisciplinary and internationally available lists. We thus did not consider country- or discipline-specific lists, which might differ in their understandings of quality, transparency, and legitimacy in academic publishing.

### Conclusions.

The lack of a clear conceptual foundation of predatory journals limits the meaning and applicability of current research on predatory journals. Our study indicates that the blacklists and whitelists examined are helpful to inform researchers about journals that are likely fraudulent or likely legitimate. However, the lists tend to emphasize easily verifiable criteria, which are easier for journals to meet, whereas dimensions that are more difficult to assess, such as peer review, are less well covered. Finally, our study illustrates the overlap between blacklists and whitelists, indicating that some journals are misclassified and that others operate in a gray zone between fraud and legitimacy. Future research should aim at better defining this gray zone. We also encourage research to further investigate the concepts of quality, transparency, and legitimacy as well as best practices in academic publishing, specifically with regard to peer review.

## MATERIALS AND METHODS

We used a mixed-methods approach, combining quantitative and qualitative methods. Using record linkage methods, we compared blacklists and whitelists in terms of overlap, i.e., with regard to the journals and publishers that they indexed. We then qualitatively examined and interpreted the inclusion criteria of blacklists and whitelists.

### Selection of blacklists and whitelists.

We searched for blacklists and whitelists in February 2018 using Google and Google Scholar. We used the search terms “blacklist,” “whitelist,” “predatory journal,” and “predatory publisher.” We selected lists that were multidisciplinary; that is, they included journals from different academic disciplines, were commonly used in studies on predatory publishing, and were accessible either free of charge or for a fee. Two authors (M. Strinzel and A. Severin) independently screened selected lists for suitability. The inclusion criteria of blacklists and whitelists were obtained from the respective websites in February and March 2018; the journals and publishers indexed in these lists were downloaded in December 2018.

### Quantitative analysis of contents.

**FIG 7:**
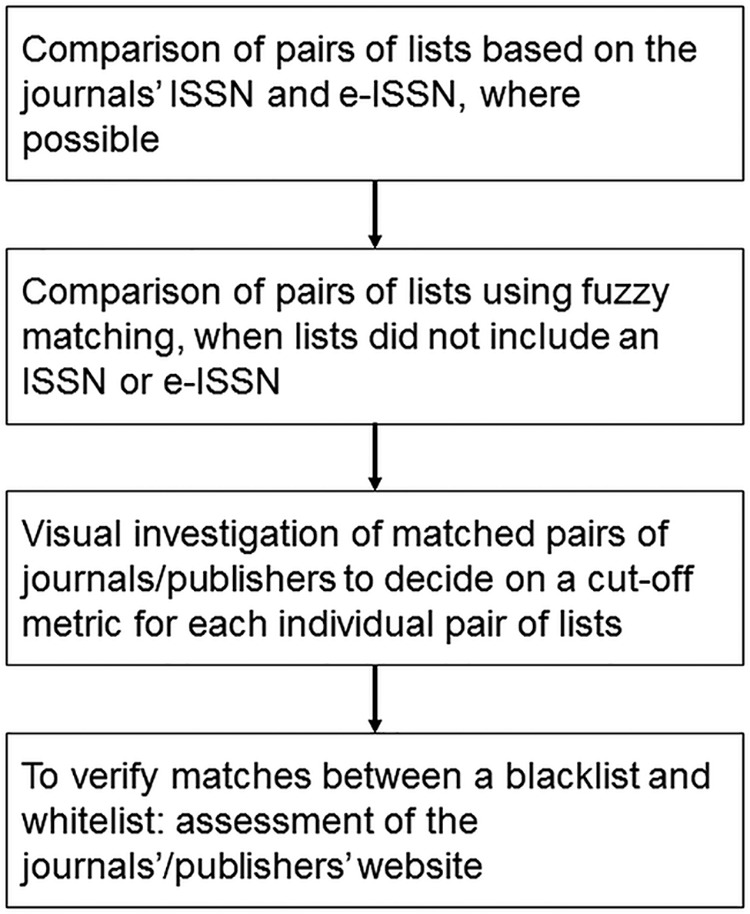
Procedure of the quantitative comparison of blacklists and whitelists.

In the first part of the study, we compared contents of lists quantitatively in terms of the journals and publishers that they include. Where possible, we compared lists based on the unique journal identifier, i.e., its ISSN or its electronic version (e-ISSN). Since Beall’s list and Cabell’s blacklist did not include an ISSN or e-ISSN for every journal, comparisons were based on the names of journals. Due to potential typographical errors and other orthographic differences between the lists, we matched strings based on their similarity, using the Jaro-Winkler algorithm in R package RecordLinkage (32). The algorithm involves computing string lengths, the number of common characters in the two strings, and the number of transpositions (33). The Jaro-Winkler metric is scaled between 0 (no similarity) and 1 (exact match). The metric was calculated for all possible pairs of journals. We chose the cutoff metric individually for each pair of lists, depending on the similarity of lists (e.g., the more orthographically similar, the higher the cutoff value). We then inspected the pairs above the cutoff score to determine whether journal names matched. For matching journal names of a blacklist and a whitelist, we further compared the journals’ publishers and websites to exclude cases where two journals were merely named the same but were from different outlets. We used Venn diagrams to illustrate the overlap between different lists. See Fig. 7 for a schematic representation of the steps used in the quantitative analysis. The procedure was repeated for publishers indexed in the four lists.

### Qualitative analysis of inclusion criteria.

In the second part of the study, we qualitatively analyzed inclusion criteria of blacklists and whitelists. Aiming to generate a more in-depth understanding of quality standards for scholarly journals employed by these lists, we conducted a thematic analysis of the inclusion criteria as stated in the lists’ formal guidelines. Thematic analysis is a technique for analyzing qualitative data, which involves the organization and description of data by examining themes within those data, thereby enabling the identification of implicit and explicit ideas (34). We conducted the analysis in three steps. First, we read and reread the sets of formal inclusion criteria and repeatedly coded their topic, that is, the aspect of a journal or publishing practice to which each criterion referred, until saturation across topics was reached (35, 36). Second, we identified and analyzed broader concepts addressed by the inclusion criteria. Aiming to facilitate a holistic understanding of the topics addressed by the inclusion criteria, we adopted a more abstract level of analysis and assessed to which dimensions of quality the inclusion criterion related. This involved an in-depth interpretation of inclusion criteria and their topics, followed by comparisons of topic frequencies across lists. Coding categories, hence, emerged from the analyses and were not developed *a priori*. In a third step, we assessed the ease of verifying criteria, with regard to the degree of subjective judgment that was required to verify whether a criterion was met, as well as to the number of sources that had to be consulted. We categorized the verifiability of inclusion criteria as follows: (i) easy verifiability, where a criterion could be verified based on an easily accessible source and without involving individual judgement; (ii) intermediate verifiability, where the consultation of several sources or contact with the journal (but no subjective judgement) was required; and (iii) difficult verifiability, where the verification of a criterion required subjective judgment. Table 6 illustrates the classification of verifiability.

**TABLE 6:** Verifiability of criteria

Verifiability status	Description	Examples of criteria
Easy	Only one source must be consulted in order to verify the criterion; no subjective judgement is required	ISSNs should be clearly displayed (DOAJ)
		The publisher displays prominent statements that promise rapid publication and/or unusually quick peer review (Cabell’s blacklist)
Intermediate	Several sources must be consulted or contact with the journal/publisher is required in order to verify the criterion; no subjective judgement is required	The publisher makes unauthorized use of licensed images on their website, without permission or licensing from the copyright owners (Beall’s list)
		The journal does not indicate that there are any fees associated with publication, review, submission, etc., but the author is charged a fee after submitting a manuscript (Cabell’s blacklist)
Difficult	Subjective judgement is required in order to verify the criterion	Articles published in the journal must be relevant to current priorities in its field and be of interest to the academic community (Cabell’s whitelist)
		The publisher dedicates insufficient resources to preventing and eliminating author misconduct (Beall’s list)

The analysis was conducted by two assessors (M. Strinzel and A. Severin), who independently repeated the steps, revised concepts, and subsequently finalized them by consensus. M. Strinzel is a linguist by training, and A. Severin is a social scientist. In cases where the two assessors assigned different categories, the inclusion criteria were discussed and a consensus reached. Throughout the process, one of the assessors (A. Severin) was blind to the lists from which the criteria originated.
