# Erratum for Strinzel et al., “Blacklists and Whitelists To Tackle Predatory Publishing: a Cross-Sectional Comparison and Thematic Analysis”

**DOI:** 10.1128/mBio.03108-20

**Published:** 2021-01-05

**Authors:** Michaela Strinzel, Anna Severin, Katrin Milzow, Matthias Egger

**Affiliations:** a Swiss National Science Foundation, Bern, Switzerland; b Institute of Social and Preventive Medicine (ISPM), University of Bern, Bern, Switzerland

## ERRATUM

Volume 10, no. 3, e00411-19, 2019, https://doi:10.1128/mBio.00411-19. Table 3 should have appeared as shown here.

**TABLE 3:** List of names of journals and publishers included in a blacklist and a whitelist^*a*^

Journal/publisher (ISSN)
Journals included in Beall’s list, the DOAJ, and Cabell’s blacklist
Ecoforum (2344-2174)
European Chemical Bulletin (2063-5346)
Global Journal of Medicine and Public Health (2277-9604)
International Archives of Medicine (1755-7682)
International Journal of Mosquito Research (2348-7941)
Journal of New Sciences (2286-5314)

Journals included in Beall’s list, the DOAJ, and Cabell’s whitelist
International Journal of Nanomedicine (1178-2013)

Journals included in Beall’s list and the DOAJ
Archives of Clinical and Experimental Surgery (2146-8133)
Asian Pacific Journal of Tropical Disease (2222-1808)
Australasian Medical Journal (1836-1935)
Canadian Journal of Biotechnology (2560-8304)
Cumhuriyet Science Journal (1300-1949)
Estudos de Psicologia (Campinas) (0103-166)
Indian Journal of Advances in Chemical Science (2320-0928)
International Journal of Advanced and Applied Sciences (2313-3724)
International Journal of Advances in Applied Mathematics and Mechanics (2347-2529)
International Journal of Business and Social Research (2164-2540)
International Journal of Development and Sustainability (2186-8662)
International Journal of Humanities and Cultural Studies (2356-5926)
International Journal of Pediatrics (2345-5047)
International Journal of Physiotherapy (2349-5987)
International Journal of Psychology and Educational Studies (2148-9378)
International Journal of Science Culture and Sport (2148-1148)
International Review of Social Sciences and Humanities (2248-9010)
Journal of Advanced Veterinary and Animal Research (2311-7710)
Journal of Animal and Plant Sciences (2071-7024)
Journal of Arts and Humanities (2167-9045)
Journal of Clinical and Analytical Medicine (1309-0720)
Journal of Coastal Life Medicine (2309-5288)
Journal of Evidence Based Medicine and Healthcare (2349-2562)
Journal of HerbMed Pharmacology (2345-5004)
Journal of IMAB (1312-773X)
Journal of Intercultural Ethnopharmacology (2146-8397)
Journal of Media Critiques (2056-9793)
Jundishapur Journal of Health Sciences (2252-021X)
Junior Scientific Researcher (2458-0341)
Mediterranean Journal of Chemistry (2028-3997)
Mediterranean Journal of Modeling and Simulation (2335-1357)
OIDA International Journal of Sustainable Development (1923-6654)
Progress in Physics (1555-5534)
Tropical Plant Research (2349-1183)

Journals included in Cabell’s blacklist and the DOAJ
Advances in Bioscience and Clinical Medicine (2203-1413)
Advances in Language and Literary Studies (2203-4714)
Advances in Science, Technology and Engineering Systems (2415-6698)
Atlas Journal of Biology (2158-9151)
Global Journal of Cardiovascular and Cerebrovascular Diseases (2309-3374)
Global Journal of Geriatrics Nursing (2309-303X)
Global Journal of Hospital Administration (2309-3609)
Global Journal of Integrated Chinese Medicine and Western Medicine (2308-6025)
Global Journal of Nursing Research (2309-2963)
Global Journal of Psychological Research (2330-913X)
Global Journal of Traditional Medicine (2308-5665)
ICTACT Journal on Communication Technology (0976-0091)
ICTACT Journal on Image and Video Processing (0976-9099)
ICTACT Journal on Soft Computing (0976-6561)
International Journal of Applied Linguistics and English Literature (2200-3592)
International Journal of Comparative Literature and Translation Studies (2202-9451)
International Journal of Education and Literacy Studies (2202-9478)
International Journal of Pharmacological Research (2277-3312)
Journal of Education in New Century (2372-6539)
Journal of Men’s Health (1875-6859)
Journal of Proteins and Proteomics (0975-8151)
Journal of Systemics, Cybernetics and Informatics (1690-4524)
Leonardo Electronic Journal of Practices and Technologies (1583-1078)
Leonardo Journal of Sciences (1583-0233)
Open Journal for Educational Research (2560-5313)
Open Journal for Sociological Studies (2560-5283)
Problems of Management in the 21st Century (2029-6932)
BJ Kines-National Journal of Basic & Applied Sciences (2231-6140)
Journal of Baltic Science Education (1648-3898)
Problems of Education in the 21st Century (1822-7864)
Problems of Psychology in the 21st Century (2029-8587)

Publishers included in Beall’s list, the DOAJ, and Cabell’s blacklist
Academia Publishing
AcademicDirect Publishing House
Atlas Publishing, LP
Australian International Academic Centre
ICTACT Journals
Insight Medical Publishing (OMICS International)
International Institute of Informatics and Systemics
Scholar Science Journals
Scientia Socialis
New Century Science Press

Publishers included in Cabell’s blacklist and Cabell’s whitelist
i-manager publications

Publishers included in Beall’s list and the DOAJ
AgiAl Publishing House
Eurasian Publications
Herald Scholarly Open Access
Hilaris
Ivy Union Publishing
Longdom Publishing
PiscoMed Publishing
Scholarly Research Publisher
Science and Education Centre of North America
Scientia Ricerca
Elewa BioSciences
International Foundation for Research and Development (IFRD)
International Academy of Ecology and Environmental Sciences
Smart Science & Technology LLC
EconJournals
Science Park Research Organization and Counselling LTD
Applied Science Innovations Private Limited
Frontiers Media S.A.
NobleResearch Publishers

Publishers included in Cabell’s blacklist and the DOAJ
B J Medical College
Innovative Journal Solutions
International Medical Society
The Dougmar Publishing Group, Inc.
Academy of Business and Retail Management
Center for Open Access in Science
Deuton-X Ltd.
Association of Educational and Cultural Cooperation Suceava from Stefan cel Mare Universit
Regional Institute of Health and Family Welfare
ASTES Publishers
Sunblo Learning Center
Serials Publications/International Science Press

a Data are as of December 2018.

