# Correction for Strinzel et al., “Blacklists and Whitelists To Tackle Predatory Publishing: a Cross-Sectional Comparison and Thematic Analysis”

**DOI:** 10.1128/mbio.01305-22

**Published:** 2022-05-23

**Authors:** Michaela Strinzel, Anna Severin, Katrin Milzow, Matthias Egger

**Affiliations:** a Swiss National Science Foundation, Bern, Switzerland; b Graduate School of Health Sciences, University of Bern, Bern, Switzerland; c Institute of Social and Preventive Medicine (ISPM), University of Bern, Bern, Switzerland

## AUTHOR CORRECTION

Volume 10, no. 3, e00411-19, 2019, https://doi.org/10.1128/mBio.00411-19. Cabells Scholarly Analytics informed us that the blacklist of predatory journals (now called Predatory Reports [https://www2.cabells.com/ {accessed 2 December 2018}]) that we downloaded at the end of 2018 and used for our analysis had been amended to correct several internal errors. The amended list consisted of 10,324 unique journals and 449 unique publishers, 347 fewer journals and 24 fewer publishers than in the original blacklist. We repeated the analyses described in our paper using this amended list to quantify overlaps in contents between blacklists and passlists. Out of the 37 journals in the intersection of Cabells Predatory Reports and the passlist of the Directory of Open Access Journals (DOAJ), 11 journals were no longer included in the Cabells amended blacklist (Fig. 1). Similarly, five publishers were no longer included (Fig. 2). Table 1 provides the names of those journals and publishers. The updated analysis thus reduced the number of journals and publishers included in both blacklists and passlists from 72 to 61 and from 42 to 37, respectively.

**FIG 1:**
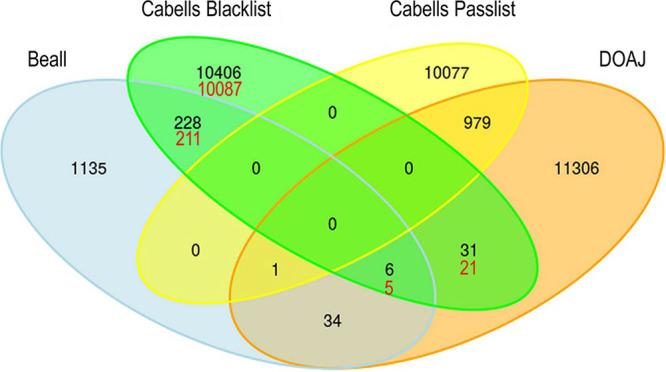
Venn diagram of journal overlap among Beall’s list, the Cabells blacklist, the DOAJ, and the Cabells passlist. The numbers of journals from the original analysis are shown in black, and the numbers that have changed in the updated analysis are shown in red. Both data sets are from December 2018.

**FIG 2:**
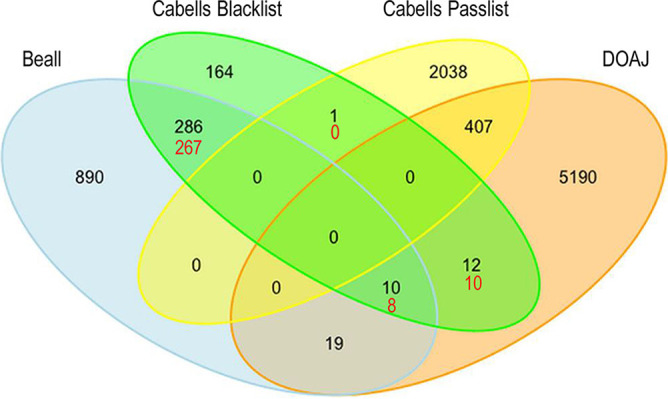
Venn diagram of publisher overlap among Beall’s list, the Cabells blacklist, the DOAJ, and the Cabells passlist. The numbers of journals from the original analysis are shown in black, and the numbers that have changed in the updated analysis are shown in red. Both data sets are from December 2018.

**TABLE 1:** Journals and publishers erroneously included in Cabells Predatory Reports (formerly Cabells blacklist) in December 2018 and included in Table 3 of our original paper

Journal	Publisher	Information in Table 3 of original paper
*Global Journal of Medicine and Public Health*	Regional Institute of Health and Family Welfare	Journal included in Beall’s list, DOAJ, and Predatory Reports (Cabells blacklist)
		Publisher included in DOAJ and Predatory Reports (Cabells blacklist)
*Advances in Language and Literary Studies*		Journal included in DOAJ and Predatory Reports (Cabells blacklist)
*Atlas Journal of Biology*		Journal included in DOAJ and Predatory Reports (Cabells blacklist)
*ICTACT Journal on Communication Technology*	ICTACT Journals	Journal included in DOAJ and Predatory Reports (Cabells blacklist)
		Publisher included in DOAJ, Beall’s list, and Predatory Reports (Cabells blacklist)
*ICTACT Journal on Image and Video Processing*	ICTACT Journals	Journal included in DOAJ and Predatory Reports (Cabells blacklist)
		Publisher included in DOAJ, Beall’s list, and Predatory Reports (Cabells blacklist)
*ICTACT Journal on Soft Computing*	ICTACT Journals	Journal included in DOAJ and Predatory Reports (Cabells blacklist)
		Publisher included in DOAJ, Beall’s list, and Predatory Reports (Cabells blacklist)
*International Journal of Comparative Literature and Translation Studies*		Journal included in DOAJ and Predatory Reports (Cabells blacklist)
*Journal of Men’s Health*	The Dougmar Group	Journal included in DOAJ and Predatory Reports (Cabells blacklist)
		Publisher included in DOAJ and Predatory Reports (Cabells blacklist)
*Leonardo Electronic Journal of Practices and Technologies*	AcademicDirect Publishing House	Journal included in DOAJ and Predatory Reports (Cabells blacklist)
		Publisher included in DOAJ, Beall’s list, and Predatory Reports (Cabells blacklist)
*Leonardo Journal of Sciences*	AcademicDirect Publishing House	Journal included in DOAJ and Predatory Reports
		Publisher included in DOAJ, Beall’s list, and Predatory Reports (Cabells blacklist)
*Journal of Baltic Science Education*		Journal included in DOAJ and Predatory Reports (Cabells blacklist)
	i-manager Publications	Publisher included in Journalytics (Cabells whitelist) and Predatory Reports (Cabells blacklist)

The amendments made to the Cabells December 2018 blacklist do not alter the interpretation of our study. As discussed in our paper, the overlaps between the blacklists and passlists indicate that some journals may operate in a gray zone for extended periods, meeting some blacklist and some passlist criteria. Furthermore, they could be “false positives” on the blacklists, i.e., journals wrongly classified as predatory. Journals on the passlists could be “false negatives,” i.e., falsely classified as legitimate based on criteria that are easy to meet but overlooking other practices, e.g., the lack of adequate peer review. Unfortunately, widely accepted and operationalized criteria for predatory and legitimate journals are presently lacking. Finally, the status of a journal or publisher can change over time. Lists, therefore, need to be kept up-to-date, and journals should be reassessed at regular intervals. The present reanalysis illustrates that our article showed only a snapshot in time. Unfortunately, we were not able to update our study to the year 2022: the costs of the Cabells data have increased 10-fold, preventing another purchase of their data.

